# Comparison of a novel microcrystalline tyrosine adjuvant with aluminium hydroxide for enhancing vaccination against seasonal influenza

**DOI:** 10.1186/s12879-017-2329-5

**Published:** 2017-03-27

**Authors:** M. D Heath, N. J. Swan, A. C. Marriott, N. J. Silman, B. Hallis, C. Prevosto, K. E. Gooch, M. A. Skinner

**Affiliations:** 1 Allergy Therapeutics Ltd, Dominion Way, West Sussex, BN14 8SA UK; 2 Public Health England, PHE Porton, Porton Down, Salisbury, SP4 0JG UK; 3 Present address: Kings College, Guys Campus, London, SE1 3QD UK

**Keywords:** Vaccine, Influenza, Adjuvant, Microcrystalline tyrosine, Aluminum, Ferret

## Abstract

**Background:**

Vaccination against seasonal influenza strains is recommended for “high risk” patient groups such as infants, elderly and those with respiratory or circulatory diseases. However, efficacy of the trivalent influenza vaccine (TIV) is poor in many cases and in the event of an influenza pandemic, mono-valent vaccines have been rapidly developed and deployed. One of the main issues with use of vaccine in pandemic situations is the lack of a suitable quantity of vaccine early enough during the pandemic to exert a major influence on the transmission of virus and disease outcome. One approach is to use a dose-sparing regimen which inevitably involves enhancing the efficacy using adjuvants.

**Methods:**

In this study we compare the use of a novel microcrystalline tyrosine (MCT) adjuvant, which is currently used in a niche area of allergy immunotherapy, for its ability to enhance the efficacy of a seasonal TIV preparation. The efficacy of the MCT adjuvant formulation was compared to alum adjuvanted TIV and to TIV administered without adjuvant using a ferret challenge model to determine vaccine efficacy.

**Results:**

The MCT was found to possess high protein-binding capacity. In the two groups where TIV was formulated with adjuvant, the immune response was found to be higher (as determined by HAI titre) than vaccine administered without adjuvant and especially so after challenge with a live influenza virus. Vaccinated animals exhibited lower viral loads (as determined using RT-PCR) than control animals where no vaccine was administered.

**Conclusions:**

The attributes of each adjuvant in stimulating single-dose protection against a poorly immunogenic vaccine was demonstrated. The properties of MCT that lead to the reported effectiveness warrants further exploration in this and other vaccine targets - particularly where appropriate immunogenic, biodegradable and stable alternative adjuvants are sought.

**Electronic supplementary material:**

The online version of this article (doi:10.1186/s12879-017-2329-5) contains supplementary material, which is available to authorized users.

## Background

Influenza A virus infections (IAV) in humans have been described for well over 100 years and certainly long before the major pandemic that occurred in 1918 with the H1N1 strain of virus [1]. Influenza viruses comprise an RNA genome which is formed of 8 different segments, thus providing ample opportunity for segments to be easily transferred between different virus strains [2]. This transfer or reassortment brings about the phenomenon of antigenic shift where influenza viruses undergo a major change in their antigenic structure as a consequence of segment-swapping and are able to transmit freely within an essentially immunologically naïve population. More limited changes in the influenza virus antigenic structures occur as a result of antigenic drift; this occurs as a consequence of minor amino acid substitutions which result from transcription and translation errors of the RNA genome [3]. The mutation rate was determined for the recent H1N1 2009 pandemic virus across two influenza seasons and was found to be 10^−3^ per nucleotide site per year [4].

The first of these factors (antigenic shift) is the key property of the virus which is considered during pandemic preparedness, however at least two of these other four ‘signature’ factors should be further investigated, these are, the higher observed transmissibility and the higher mortality in younger populations [3]. This was exactly the scenario with the recent pandemic caused by the “new” H1N1 virus in 2009 where infection with pandemic influenza virus resulted in a range of symptoms in different people which varied from mild, sub-clinical infection to severe viral pneumonia requiring hospitalisation and specialist intensive care [5]. The increased transmissibility is almost certainly a result of the emergence of an effectively “new” virus by antigenic shift which is then able to infect a naïve population; younger members of the population being more likely to be immunologically naïve having never been exposed to similar viruses, whereas older subjects may be more protected due to immunological memory [6].

Because of these constant evolutionary changes, leading to antigenically novel strains and subtypes emerging within the human population, seasonal vaccines against IAV viruses have to be updated on an annual basis. A recent study on the effectiveness of influenza vaccination indicated that effectiveness of the vaccine ranged from 36% to 58% with greater effectiveness against H1N1 strains than H3N2 viruses [7]. Indeed, recent data indicate that during the winter of 2014–15 in the Northern Hemisphere, the H3N2 circulating virus strains were significantly different from the vaccine strain and resulted in much lower or zero effectiveness against these strains [8]. One approach to the improvement of efficacy of influenza vaccines has been the evaluation of adjuvants to promote both improved immunogenicity as well as dose sparing in pandemic situations.

Seasonal influenza vaccines are widely produced and used and are formulated as a trivalent (A/H1N1, A/H3N2 and B), formalin-inactivated and split virus preparation, typically containing 15 μg of each haemagglutinin (HA) protein in a standard adult dose. Although widely used, the trivalent influenza vaccine (TIV) is known to have relatively poor and variable effectiveness [9]. In the naïve ferret model, non-adjuvanted TIV shows poor efficacy against intra-nasal challenge with homologous virus. Ferret vaccine efficacy studies have typically used the standard adult human dose in each ferret, either as two or more doses [10, 11], or formulated with adjuvant [12, 13].

For almost a century, salts of aluminium (hydroxide and phosphate) were the only approved adjuvants in humans [14]. An often described limitation of aluminium adjuvants relates to the non-biodegradable nature and the stimulation of so-called T-helper type 2 (Th2) as opposed to Th1 immune responses, which affect the type and quality of antibody responses produced [15]. Therefore the goals of new adjuvants in combination with an influenza target vaccine, are (i) to facilitate recognition of the antigen, thereby facilitating the use of smaller doses (dose sparing) of antigen (ii) to be biodegradable and biocompatible, (iii) to be without toxic or inflammatory side effects, (iv) to trigger protective Th1-like immune responses as well as antigen-neutralising antibodies, thereby increasing the proportion of subjects that become protectively immunised and (v) increase seroconversion rates in populations with reduced responsiveness (i.e. infants and the elderly).

Aluminium hydroxide has been used as a depot candidate in many formulations to date including influenza vaccine candidates [16, 17], however, it could be limited as an influenza candidate for which annual vaccination may favour the use of a depot adjuvant with biodegradable properties ensuring clearance prior to revaccination, in addition to adjuvant candidates which stimulate more effective Th1 T-cell responses [18]. Allergy Therapeutics (AT) has pioneered the concept of a slow-release licensed depot adjuvant formulation for allergy vaccines with a proven safety and efficacy profile [19, 20]. Whereas other manufacturers still use mainly aluminium hydroxide (alum), AT uses Micro-Crystalline Tyrosine (MCT), a natural amino acid formulation, in its vaccine formulations. MCT exhibits a high adsorptive power for proteins at neutral pH, it enhances the induction of IgG antibodies with no unusual propensity to stimulate IgE, has a half-life of 48 h at the site of injection while delivering a sustained release of antigens for prolonged immune exposure and, unlike alum, it is fully metabolised within the body [19, 21, 22]. Its mechanistic pathways as an adjuvant are currently sought and an ongoing study has recently highlighted induction of specific T cell responses. Measurements investigating specific T cell responses, DC activation and expression markers in challenge models are ongoing (Prof. Thomas M. Kuendig, University of Zurich, personal communication, June 2016). Moreover, MCT offers a compatible mode of adsorption with 2nd generation immunomodulators (i.e. TLR agonists). As such, has been combined successfully with Monophosphoryl Lipid A (MPL) which offers a novel ultra-short-course allergy immunotherapy as a named patient product [23], of which, has recently completed a successful phase II dose ranging trial in Europe [24], while different iterations (allergy indications) of the platform are in current clinical development both in the EU and US. MPL is a TLR-4 agonist able to modulate Th1 immune reactivity, its physicochemical and biological compatibility with MCT has been described previously [19–22].

The aims of this study were to compare the efficacy of TIV with alum (Alhydrogel) to TIV with MCT in protecting ferrets against a low-dose challenge with a clinically relevant human H1N1 virus. The low-dose challenge model has been shown to be more sensitive in demonstrating antiviral activity, while at the same time producing a larger amount of virus shedding and more representative disease kinetics when compared to the traditional high (10^6^ pfu) dose ferret challenge model [25].

## Methods

### Virus and vaccines

Influenza A/California/04/09 virus (H1N1) was propagated in MDCK cells. Viral genomic RNA from the virus stock was fully sequenced and showed no differences from the published A/California/04/09 sequence. The vaccine used was Inactivated Influenza Vaccine (Split Virion) BP from Sanofi Pasteur, containing antigens from the viruses recommended for the 2014–15 season. Each 0.5 ml dose contained 45 μg HA protein, 15 μg each from the H1N1, H3N2 and B virus components. The H1N1 component was from the A/California/07/09-derived vaccine strain NYMC X-179A, which is antigenically indistinguishable from the challenge strain. The other components were H3N2 (A/Texas/50/12-like) and B/Massachusetts/2/12.

### Adjuvants

Alhydrogel (2% *w*/*v* suspension of aluminium hydroxide; Invivogen, USA) was mixed with vaccine in the ratio 43 μl Alhydrogel per 1 ml vaccine plus 1 ml buffered saline, pH 6, containing 0.5% *w*/*v* phenol. Micro-crystalline tyrosine (MCT) was manufactured at Allergy Therapeutics Ltd, Worthing, UK, as a 4% *w*/*v* suspension in buffered saline, pH 6, containing 0.5% *w*/*v* phenol and was mixed 1.05:1 by volume with vaccine (2% target concentration). For both adjuvants, the suspension was mixed at room temperature for 1 h prior to vaccination.

### Sample preparation; MCT adsorption capacity

300 μl of 100 μg/ml H1N1 antigen (Influenza A H1N1 (A/Puerto Rico/8/1934), Haemagglutinin from SinoBiologicals Inc. was mixed with 700 μl of 2%*w*/*v* tyrosine blank (MCT) for 1 h at room temperature, to give a target H1N1 concentration of 30 μg/mL, followed by centrifugation of the sample for 4 min at 3 x *g*. An identical process was followed to produce the two controls, one control (control A) comprised antigen +DPBS and a second control (Control B) contained MCT alone. Both control groups are representative of Group A and Group B vaccines used in this study.

The protein concentration present in the supernatant from the sample and both controls were determined using Bradford reagent [26], with the exception that the standard curve was prepared in DPBS to remove any possibility of interference from different buffers.

**Fig. 1:**
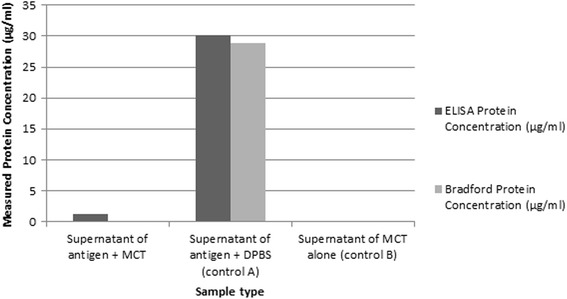
Total and specific protein concentrations of H1N1 measured by ELISA and Bradford reagent

ELISA analysis confirmed >95% adsorption of the antigen by MCT (Fig. 1).

### ELISA analysis

The supernatant from the sample and controls prepared as described in MCT adsorption capacity, were diluted 1:10,000, according to the manufacturer’s instructions (Sino biological Inc.). The supernatant from the MCT control (control B) was loaded undiluted. Sample and controls were analysed as per the manufacturer’s instructions.

In summary, polystyrene microplates were pre-coated with a mouse monoclonal antibody against H1N1, the plate was washed. A standard curve of H1N1 protein was prepared covering the range 46-3000 pg/ml and the samples loaded on to the ELISA plate. The plate was incubated for 2 h at room temperature then washed. The detection antibody was added and the plate incubated for a further 1 h at room temperature. The plate was washed and substrate solution added. The plate was incubated for a further 20 min and stop solution was added and the UV absorbance was analysed using a plate reader (Envision, Perkin-Elmer) at 450 nm. The concentrations of samples was analysed with comparison to the results obtained for the standard curve.

### Ferret study

Eighteen ferrets (*Mustela putorius furo*) were obtained from Highgate Farm, with starting weights between 0.78–1.79 kg. The experimental animal work described here was scrutinized and approved by the Animal Welfare and Ethical Review Body of Public Health England (Porton), as required by the UK Home Office Animals (Scientific Procedures) Act, 1986. The premises in which the work was conducted are approved under Home Office Certificate of Designation PCD70/1707. A serum sample from each animal was screened for absence of antibodies to influenza A virus by HAI prior to commencement of the study. Animals received an intra-muscular vaccination of 1 ml total volume, divided equally between the two hind legs. Twenty-one days later, sedated ferrets were challenged by intra-nasal instillation of 0.2 ml virus, containing 100 PFU A/California/04/09, divided between the two nares. Nasal wash liquids were collected daily thereafter, using 2 ml PBS per ferret. The cell content of the nasal wash fluid was determined by haemocytometry. Animals were euthanised 5 or 10 days post-challenge for viral and histological tissue analysis.

### Serum antibodies

Influenza H1N1-specific antibodies were titrated by haemagglutination inhibition (HAI), and neutralizing antibodies were titrated by microneutralization (MN) as described elsewhere [27].

### Virus load

Infectious virus in nasal wash fluid was determined by plaque assay on MDCK cells. Respiratory tract tissues were collected into RNAlater solution (Sigma-Aldrich, UK) for RNA extraction. Quantification of the extracted viral M gene RNA was performed using real-time qRT-PCR, employing a synthetic T7 transcript of the A/California/04/09 M gene as a standard of known copy-number [25].

### Statistical methods

Non-parametric tests (Mann-Whitney U-test) and 1-way ANOVA were performed using Minitab 16 software. Tests were considered statistically significant where *p* ≤ 0.05.

## Results

### Antibody responses to vaccination

**Fig. 2:**
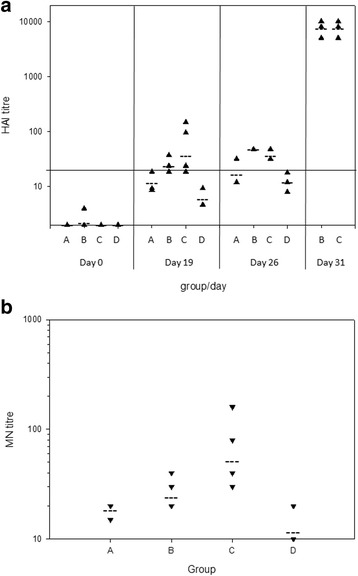
Serum HAI and MN titres to H1N1 virus. **a** For HAI test, titres below the black line (a titre of 20) were considered to be sero-negative. ▲ HAI titres of individual sera; group mean titres. **b** For MN test, pre-bleed sera gave titres of ≤10. ▼ MN titres of individual sera; group mean titres

Ferrets were divided into 4 groups for vaccination as follows: (A) TIV unmodified (*n* = 3); (B) TIV formulated with MCT (*n* = 6); (C) TIV formulated with Alhydrogel (*n* = 6); and (D) mock-vaccinated with PBS (*n* = 3). At 19 days post-vaccination serum samples were collected for determination of influenza H1N1-specific antibodies by HAI and MN tests (Fig. 2). Group mean HAI titres for groups B and C were higher than those for groups A and D on day 19, although this trend was not statistically significant. In the ferret model an HAI titre of ≥20 is widely considered to be sero-positive, as in the case of human sera [28, 29]. No ferrets in groups A or D showed sero-conversion, whereas 4 of 6 ferrets in group B and 3 of 6 ferrets in group C were sero-positive following vaccination alone. Similarly, MN titres were higher for groups B and C (Fig. 2b), although this was not significant by 1-way ANOVA (*p* = 0.085). HAI titres correlated well with the MN titres for individual ferrets (R^2^ = 0.93). Group C showed a significantly higher mean MN titre than group B (Mann-Whitney test, *p* = 0.03).

All animals were challenged with H1N1 virus via the intranasal route 21 days post-vaccination and terminal sera were collected from 3 ferrets per group on days 5 (all groups) and 10 (groups B and C only) post-challenge. HAI tests on the terminal sera showed a modest increase in group mean titres at day 26 (5 days post-infection) compared to day 19, but a much greater increase at day 31 (10 days post-infection) (Fig. 2a). Groups B and C showed significantly higher mean titres than groups A and D on day 26 (1-way ANOVA, *p* = 0.002); there was no significant difference in HAI titre between groups B and C on either day.

### Protection against influenza infection

**Fig. 3:**
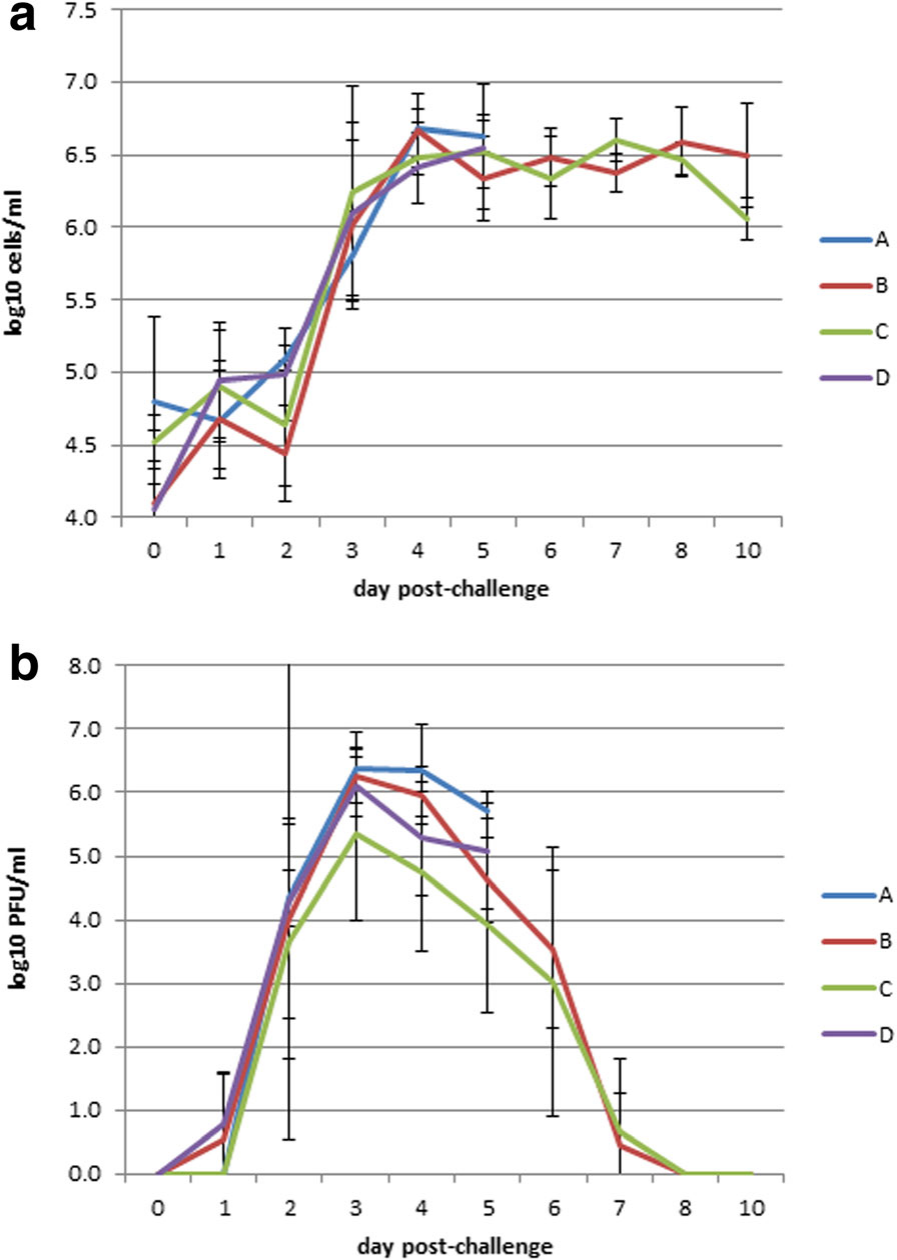
Nasal wash viable cell concentration (**a**) and infectious virus titre (**b**). Samples without detectable virus were scored as 1 PFU/ml to allow plotting

Ferrets were monitored for signs of disease following intra-nasal challenge. All challenge groups showed weight loss in the 5 days following infection, with the greatest loss in unvaccinated group D (6.3%), and the least in vaccinated group B (3.8%). The differences between groups were not statistically significant (Additional file 1). The only clinical signs that were observed were sneezing and diarrhoea. The most frequent observation of sneezing was in group D and the least in group B; again the differences between the groups were not statistically significant. A sharp rise in viable cell concentration in nasal wash fluid has been used as a surrogate marker for the host response to influenza infection [25]. All groups showed a rise in cell count between days 2 and 3 post-infection (Fig. 3a). Infectious virus shedding was monitored by plaque assay of nasal wash fluids. All ferrets were found to shed infectious virus from their nasal cavities, with a peak on day 3 post-infection (Fig. 3b). Group C showed a lower mean titre on days 2 to 5, but differences between groups were not statistically significant. Using area under the curve as an indication of total virus shedding, group C shed significantly less virus over the course of the infection than group B (Mann-Whitney test, *p* = 0.04). Virus shedding became undetectable in surviving animals by 8 days post-infection (Fig. 3b).

### Viral RNA replication in ferret tissues

**Fig. 4:**
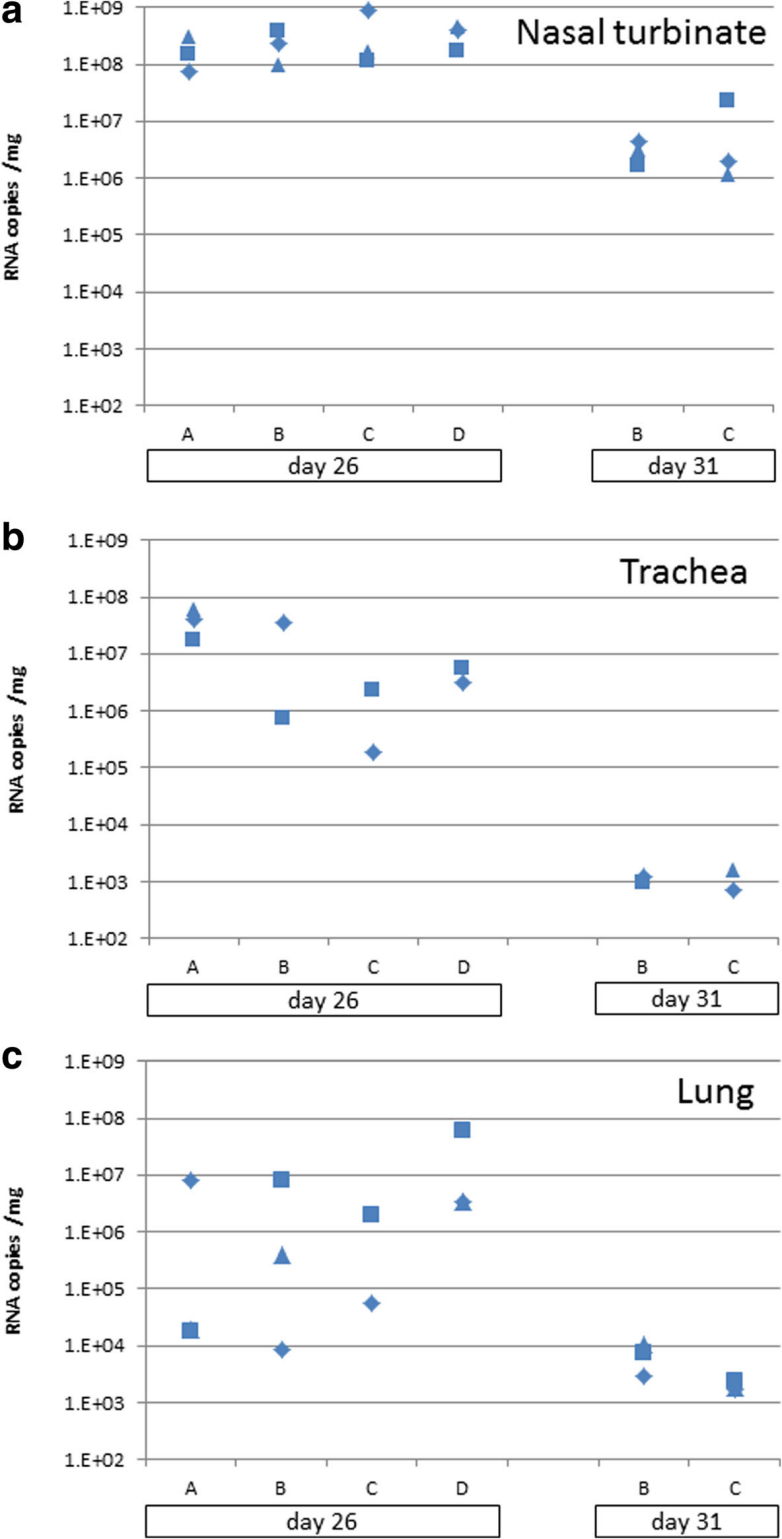
Tissue RNA loads as copies of M gene RNA per mg tissue. **a** nasal turbinate’s, **b** trachea, **c** lung. Panels a and b show titres of individual ferrets. In panel c, for each ferret 3 lung lobes were sampled and the mean was taken

RNA was extracted from nasal turbinate, trachea and 3 lobes of the lung from each animal post-mortem. Viral RNA concentration (M gene) was determined by real-time PCR and normalised for the amount of tissue extracted. As expected the vRNA levels dropped in all tissue types between days 5 and 10 post-challenge (Fig. 4). None of the vaccinated groups showed clear reductions in vRNA load in any of the tissues, relative to the unvaccinated group D, except that, when considering the combined lung samples for each animal, group A showed significantly lower vRNA loads than group D at 5 days post-challenge (Mann-Whitney test, *p* = 0.04).

## Discussion

This study provides a comparative analysis of efficacy using two established depot adjuvants in protecting against a low-dose challenge with a human H1N1 virus in a ferret model. Ferrets are considered the most representative model for studying influenza pathology since it has been extensively characterised and used for influenza vaccine development [30, 31].

Aluminium adjuvants induce robust antibody responses which make it suitable for use in vaccines that target pathogens, neutralised primarily by antibodies. Long-term success in the use of aluminium-adjuvanted vaccines targeting intracellular pathogens, in the context of protective efficacy, is less recognised and highlights the limitations in providing protective efficacy in human populations that require robust Th1 T-cell responses, as a consequence of its function to preferentially induce Th2 cells [32].

Co-precipitates of micro crystalline tyrosine (MCT) and proteins have been extensively used and endorsed by health authorities for use as a depot adjuvant in long-course allergen specific subcutaneous immunotherapy [19]. As a consequence, the use of MCT in infectious disease models and cancer is now being explored and is showing early promise [33].

The adsorption capacity of MCT was assessed independently since this adjuvant has not previously been formulated with an infectious disease target. Adsorption of infectious disease antigens to aluminium adjuvants is well documented has been shown to improve vaccine potency and stability [34]. The mode of action of an adjuvant can be influenced by the amount of antigen physically adsorbed to the adjuvant and, in some cases, considered to be an important aspect for their function [35]. As a consequence, The World Health Organisation has since recommended a figure of >95% adsorption at 0.5% antigen loading levels in some indications [36] and the results presented herein (Fig. 1) confirm >95% adsorption of MCT with the H1N1 target antigen. The aromatic ring of Tyrosine in its crystalline complex with a target offers an alternative predominant mechanism of adsorption to aluminium which is governed via ligand exchange between hydroxyl groups on the adjuvant and available phosphorylated groups of the antigen. A number of advantages may also exist in the use of MCT alone and in synergy within adjuvant system complexes. For example, in an adjuvant-adsorption study, Bell et al. (2015) characterised the compatible nature of MCT with a TLR4 agonist, inferring pi-chi interactions as a predominant mechanism of adsorption. Some TLR agonists and antigens do not readily adsorb to Aluminium, but may be more compatible with alternative formulations [37].

A single dose (45 μg) of HA protein (15 μg each from the H1N1, H3N2 and B components) adjuvanted with either MCT (study group B) or aluminium hydroxide (study group C) was effective in generating a sero-positive (HAI > 20) titre 2 days prior to challenge (19 days post-vaccination). An HAI titre of >40 is considered protective in human sera and by 26 days post-vaccination (5 days post challenge), 3/3 ferrets in group B (MCT), 1/3 ferrets in group C (Alum), and 0/3 ferrets in groups A and D showed HAI titres >40. However, 10 days post challenge a much greater increase in HAI titres was produced with no significant differences observed between the two adjuvanted groups.

Neutralizing antibody titres, determined 2 days prior to challenge, also showed a greater response in the two adjuvanted vaccine groups, B and C, compared to the non-adjuvanted group A, with alum adjuvant giving a higher mean titre. The close correlation of HAI and neutralization titres suggests that the two adjuvants were inducing functionally equivalent influenza-specific antibodies.

The intra-nasal challenge of ferrets with 100 PFU H1N1pdm09 virus has previously been used to demonstrate efficacy of both a defective influenza particle and of oral oseltamivir therapy [25, 38, 39]. It is well established that non-adjuvanted inactivated influenza vaccines perform very poorly in the ferret model [10, 11, 40], so the low-dose challenge was chosen for this study in order to minimise the possibility of the virus infection overwhelming any immunity which had been induced. While no significant protection was observed following a single dose of vaccine, in terms of virus shedding or viral replication in the lungs, both alum and MCT adjuvants clearly induced levels of influenza-specific functional antibodies prior to infection which were not observed in the non-adjuvanted vaccine group. There was also a trend towards reduced disease (weight loss and nasal symptoms) in the MCT-adjuvanted group B.

The addition of an adjuvant to an existing vaccine, as has been done for influenza [18, 41–43], represents a potential and substantial benefit, where seroconversion rates and protective antibody titres in populations with reduced responsiveness (i.e. infants and the elderly) is an issue. Selection of an appropriate adjuvant will be influenced by the type of CD4+ T cell response required for protection. In the context of available H1N1 influenza vaccine targets, there exists variable and relatively poor effectiveness which may be linked to the lack of anti-viral Th1 responses. Two existing adjuvants MF59 and AS03 have been explored in this context. MF59, an immunological adjuvant that uses squalene, has been successfully formulated in licensed versions of the influenza vaccine worldwide, with significant increase in vaccine efficacy noted in clinical trials [41, 42]. MF59 appears to be particularly effective in APC recruitment and uptake, with subsequent drainage to the lymph nodes where an appropriate immune response is induced generating robust antibody titres consistent with protective efficacy (>40 HAI). AS03 is another squalene-based adjuvant, used successfully in a pandemic H1N1 strain, again, immune responses are robust and confer levels indicative of protection, however, the persistence of the immune response particularly in infants and the elderly can differ and warrants further exploration in future vaccine candidates [43].

In a more recent study assessing adjuvant (GLA-SE; a TLR agonist) formulated with an H5N1 antigen, the authors highlighted the critical nature in the quality of CD4+ T cell responses for protection and survival. The strategy here was aimed at inducing anti-viral Th1 responses through activation of TLR4 [10].

Our study highlights the attributes of Aluminium and, for the first time in an infectious disease model, MCT, in stimulating single-dose protection against a poorly immunogenic vaccine. Antibody responses are an important component in anti-influenza protection [44, 45]. While our study demonstrated the robust HAI titre generated by both groups receiving adjuvant candidate formulations (vs. unadjuvanted), it is the quality (functionality) of this response that requires further consideration in the context of what *conditions* increased antibody production facilitates improved protection. This would need to be considered to further assess its use in this and/or other models, or as part of a “mix and match” adjuvant systems approach. MCT’s immunological (Th1; IgG) synergy with TLR mimetics has been established in allergy immunotherapy [46], while offering a unique platform for adsorption to antigen targets and/or 2nd generation immunomodulators/adjuvants, as earlier described.

While the reported effectiveness of adjuvanting an influenza target are encouraging [18, 41]; the properties of each adjuvant, alone, in the context of a human influenza vaccine target may be limiting.

However, the properties of MCT that lead to the reported effectiveness here, and elsewhere [23, 33], permits further consideration in this and other vaccine targets - especially those found to be weakly immunostimulating, non-biodegradable or those which bind poorly to existing antigens or when combined with other second generation immunomodulators/adjuvants. Further studies are now underway in different infectious disease models, while exploring the immunological signature of MCT powered to confer reproducibility.

## Conclusions

The attributes of each adjuvant in stimulating single-dose protection against a poorly immunogenic vaccine was demonstrated. The use of MCT alone or in “mix and match” adjuvant combinations for existing, new and/or emerging diseases warrants further exploration.

## Additional file

Additional file 1: Weight loss analysis. (TIFF 88 kb)

## Abbreviations

Alum: Alhydrogel adjuvant
AT: Allergy therapeutics plc
H1N1: Influenza A/California/04/09 virus
IAV: Influenza A virus vaccines
MCT: Microcrystalline tyrosine adjuvant
TIV: Trivalent influenza vaccine
WHO: World Health Organisation
