# Supplementary figures and images for: Comparison of a novel microcrystalline tyrosine adjuvant with aluminium hydroxide for enhancing vaccination against seasonal influenza
Source: BMC Infect Dis. 2017 Mar 27;17:232. doi: 10.1186/s12879-017-2329-5 (PMC5369220; doi:10.1186/s12879-017-2329-5)

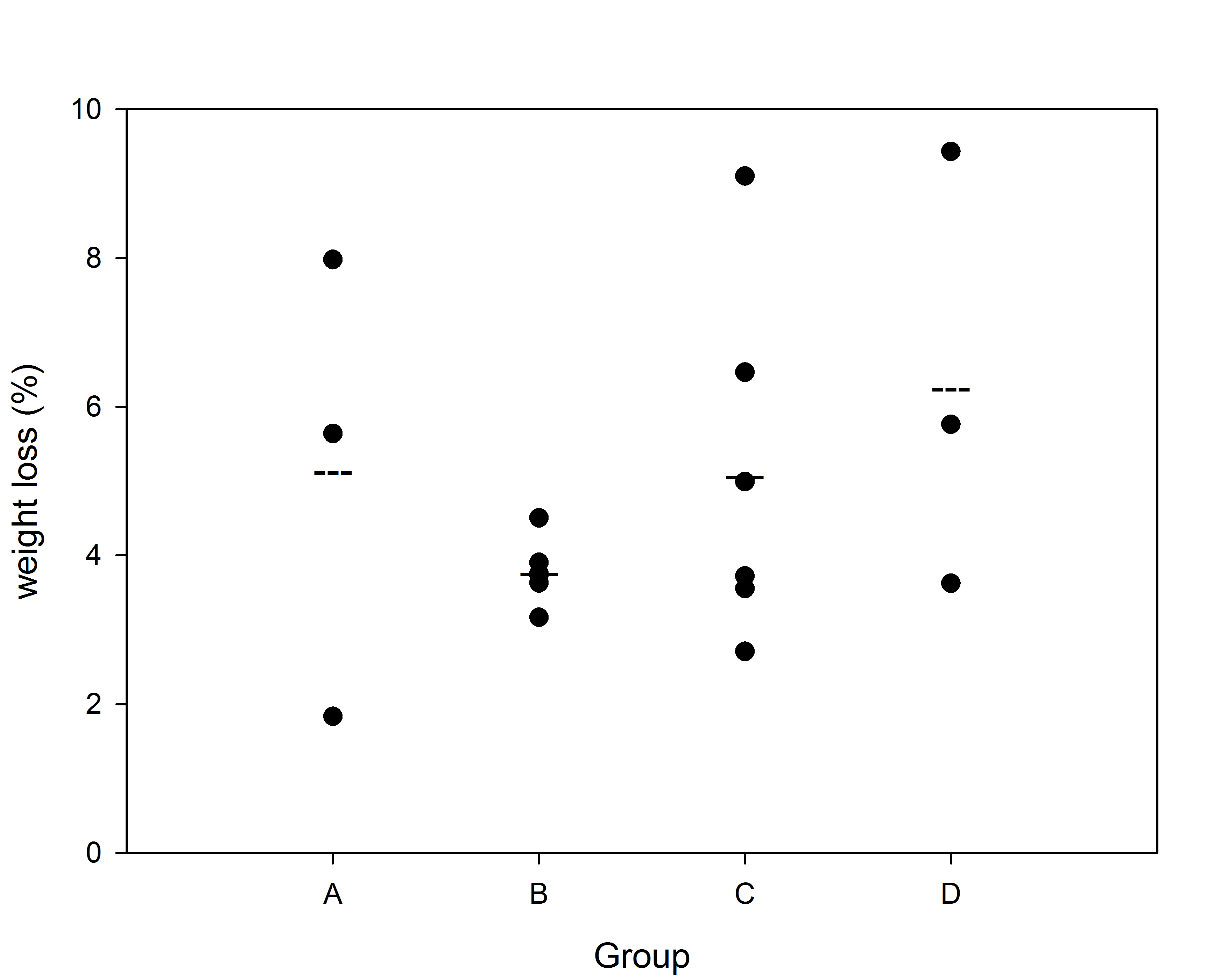

Supplement: Additional file 1: — Weight loss analysis. (TIFF 88 kb) [file 12879_2017_2329_MOESM1_ESM.tif]
